# Influence of Endoplasmic Reticulum Stress on Urokinase Plasminogen Activation

**DOI:** 10.3390/biom16091235

**Published:** 2026-08-26

**Authors:** Diana Culej Bošnjak, Doris Janjić, Petra Korać, Mariastefania Antica, Maja Matulić

**Affiliations:** 1 Department of Biology, Faculty of Science, University of Zagreb, Horvatovac 102, 10 000 Zagreb, Croatia; diana.culej2010@gmail.com (D.C.B.); djanjic1210@gmail.com (D.J.); petra.korac@biol.pmf.hr (P.K.); antica216@outlook.com (M.A.); 2 Ruđer Bošković Institute, Bijenička 54, 10 000 Zagreb, Croatia

**Keywords:** urokinase plasminogen activator, plasminogen activator inhibitor, endoplasmic reticulum stress, thapsigargin, sodium salicylate

## Abstract

The urokinase plasminogen activator or urokinase is a highly specific extracellular protease involved in numerous physiological and pathological processes. Its activity is a consequence of its interplay with its inhibitor, PAI1, and receptor, uPAR, and is finely regulated at several levels. The aim of the work was to investigate whether endoplasmic reticulum stress can modulate urokinase activity. Two tumor cell lines grown in cell culture were treated with Thapsigargin and sodium salicylate, inducers of ER stress response. Urokinase activity was determined in the conditioned media, and expression of uPA system molecules and molecules involved in response to ER stress in cell lysates was measured. ER stress influenced urokinase activity: while in the glioblastoma line its activity was increased, in breast cancer cells it was decreased. Differences in activity were a consequence of urokinase and PAI1 expression at the protein and RNA level. However, ER stress decreased cell migration, invasion, and proliferation regardless of the changes in urokinase activity. Gene expression analysis indicated that cell specific activation of some transcription factors and pathways could be responsible for different urokinase activity regulation.

## 1. Introduction

Urokinase plasminogen activator (urokinase, uPA) is a highly specific extracellular protease which activates plasminogen to produce plasmin. Plasmin participates in extracellular matrix remodeling, cell migration, and invasion, and has many important physiologic roles through tissue degradation, such as blood clot dissolution. As plasminogen is present ubiquitously in the organism, many physiological processes are regulated by its activation by plasminogen activators. The uPA system comprises urokinase, PAI1, its inhibitor, and uPAR, a membrane urokinase receptor. Urokinase is secreted from the cell in the form of prourokinase and is activated extracellularly. Its activity is regulated by uPA interactions with the urokinase inhibitor, PAI1. After urokinase and its inhibitor are bound to uPAR, the complex is internalized and degraded [1,2]. uPA system molecules are regulated on the transcriptional level, but also post-transcriptionally. It was found that the net uPA activity depends on the ratio between the uPA and PAI1 level [2]. Their transcription is regulated by multiple signaling pathways, including MAPK, PI3K-Akt, Wnt, TGF-β, and NF-κB, many of which are activated under stress conditions [3,4,5,6]. Some examples are JNK kinase pathways found to increase urokinase activity and expression in different cell types after treatment with some DNA damaging agents, disruption of cytoskeleton, treatment with phorbol esters or agents interfering with protein and mRNA metabolism, and protein phosphorylation [3,4,5,6,7,8,9]. On the other hand, it is considered that various chemotherapeutic agents and stress-inducing stimuli trigger the integrated stress response, which comprises both cytoplasmic and endoplasmic reticulum (ER) stress responses (ERS). The latter can be initiated by activation of ER receptors on the ER membrane. ER response is triggered by accumulation of unfolded proteins in the ER and a lack of free BiP chaperones. There are three ER membrane receptors whose activation can initiate unfolded protein response (UPR): IRE1α, PERK, and ATF6. Activation of PERK leads to phosphorylation of eIF2α and translational inhibition, as well as activation of transcription factors, which increase chaperone expression. IRE1α cleaves *XBP* mRNA to produce a shorter form, XBPs, which also acts as a transcription factor for chaperone production. The third pathway involves ATF6 cleavage and the transport of its cleaved form to the nucleus to influence transcription. Beside translational inhibition and production of chaperones, UPR also involves changes in lipid metabolism and can lead to autophagy and apoptosis [10,11]. It was also shown that different chemotherapeutics, amino acid starvation, hypoxia, and other stress conditions can induce UPR gene expression. Pakos-Zebrucka et al. [12] described this response as the integrated stress response, regulated through ATF4 modulation, dependent on the cellular context but also on the intensity of the stress. In our previous work we showed that different chemotherapeutics modulated urokinase activity in different cell lines, and that the cell response was often cell type specific. As it was found that some of these chemotherapeutics also induce ER stress, the aim of this work was to show whether ER stress is responsible for uPA activity modulation, and how changes in uPA activity can influence tumor cell microenvironment. We treated various tumor cell lines with Thapsigargin, a SERCA ATPase inhibitor known to induce ER stress and sodium salicylate (NaS), previously shown to modify the urokinase system. Then we analyzed the uPA system at the levels of activity, RNA, and protein expression, along with the expression of ER stress-induced molecules [13,14]. We concluded that ER stress can modify uPA activity and that the cellular response was cell type-specific.

## 2. Materials and Methods

### 2.1. Cell Culture

Human glioblastoma cell line A1235, human embryonal kidney cells HEK293, breast cancer cell line MDA MB-231, neuroglioblastoma cell line H4, and lung carcinoma cell line A2182 were grown in DMEM supplemented with 10% fetal bovine serum (both from Sigma, Marlborough, MA, USA) under standard conditions. Cell lines HEK293, MDA MB-231, HeLa, and H4 are available at ATCC (Manassas, VA, USA), while A1235 and A2182 cells were a kind gift from S. A. Aaronson (National Cancer Institute, Bethesda, MD, USA) [15]. For cell proliferation analysis, 10^4^ cells/well were seeded in 48 well plates in triplicate and treated with different concentrations of Thapsigargin. Cells were counted every 24 h. All experiments were independently performed in triplicate to ensure reproducibility. Cells were treated with Thapsigargin, epigallocatechin gallate ECGC, Icerguastat, GSK2606414 (all from MedChemExpress, Princeton, NJ, USA), and sodium salicylate (Kemika, Croatia).

### 2.2. Analysis of Enzyme Activity

uPA activity was assayed by radial caseinolysis. Cells were treated with chemotherapeutic agents in DMEM supplemented with 10% serum. After 24 h, the medium was replaced with serum-free DMEM for an additional 6 h, after which the medium was collected. This conditioned medium was then analyzed on agarose plates containing plasminogen (Sigma) and casein (Sigma) as substrates. The size of the lysis zones was compared with a calibration curve done by different concentrations of human uPA (Leo Pharmaceutical Products, Ballerup, Denmark). Relative uPA activity was normalized to the amount of protein cells lysed in a nonionic detergent lysis buffer [14,16]. Experiments were done in two biological replicates and enzymatic analysis for each was done in duplicate. Basic experiments with sodium salicylate and Thapsigargin treatment were done six times. For zymography, conditioned media were collected, concentrated with centrifugal filters (Amicon Ultra 10K, Millipore, Merck KGaA, Darmstadt, Germany) and nondenatured samples run on polyacrylamide gel. After incubation in 2.5% Triton x-100 and water, the gel was laid over the agarose gel containing casein and plasminogen and incubated on 37 °C overnight, according to protocol [16]. Protein concentration in cell lysates was determined by Bradford assay [17].

### 2.3. Western Blot Analysis

Protein cell extracts were prepared in a lysis buffer containing 1% nonionic detergent (137 mM NaCl, 10% glicerol, 1% Triton X-100, 20 mM Tris pH 7.5, 2 mM EDTA) or in RIPA lysis buffer (150 mM NaCl, 1% Triton X-100, 0.5% Na desoxycholate, 0.1% SDS, 50 mM Tris pH 8.0), with the addition of protease inhibitors (Karl Roth, Karlsruhe, Germany) [17]. The molecular weight markers used were prestained and transferred to the membrane together with the proteins and manually indicated after the film alignment with the membrane. After electrophoresis on a 10–12% SDS-PAGE gel, proteins were transferred to a PVDF membrane, blocked in nonfat dried milk (Sigma) or bovine serum albumin (Karl Roth) dissolved in TBST buffer (20 mM Tris, 150 mM NaCl with addition to up to 0.1% Tween), according to the manufacturer’s protocol. Membranes were incubated with primary antibodies specific for PAI1 (612024, Becton Dickenson, Franklin Lakes, NJ, USA), uPA (15800), ATF4 (11815), BiP (3177) (all Cell Signaling Technology, Danvers, MA, USA), and β-actin (sc69879) (Santa Cruz, Dallas, TX, USA), according to the protocols of their manufacturers. After incubation with appropriate secondary anti-rabbit and anti-mouse antibodies (Sigma-Aldrich), proteins were detected by chemiluminescence (Biorad, Hercules, CA, USA) on an X-ray film (Kodak, Rochester, NY, USA). Densitometric analysis was performed using the ImageJ program (version 1.52a, National Institute of Health, Bethesda, MD, USA). Relative expression is presented as a ratio between values obtained for each protein and those obtained for β-actin expression. Western blot analysis was done twice.

### 2.4. Migration and Invasion Assays

Cell migration was analyzed by a wound healing test. The confluent cell layer was scratched with a 100 µL-volume tip, and cells were incubated in the fresh media with or without chemotherapeutics. Microphotographs were taken immediately after scratching and after 16–24 h (Axiovert 40 CFL Zeiss with AxioCam MRm camera, Jena, Germany), and the scratch dimensions were measured in the ImageJ program. At least nine measurements were taken per sample [18].

For the invasion assay, 5–7 × 10^4^ cells were seeded on a transwell chamber membrane (Brand, Wertheim am Mein, Germany) coated with MaxGel ECM (Sigma-Aldrich, USA). MaxGel was prepared according to the manufacturer’s protocol. Cells were seeded in DMEM without serum, with or without the tested drug, and were allowed to migrate toward DMEM with 10% bovine fetal serum, supplemented with the same drug concentration. After 16–24 h incubation, membranes were fixed and stained with crystal violet and microphotographed with a stereo microscope (Stemi 2000-C, Zeiss, Jena, Germany), and the cells were counted in the ImageJ program [19]. Experiments were done in two biological replicates.

### 2.5. DNA PAGE Electrophoresis

DNA was run on a 10% polyacrylamide gel in 1× TBE buffer, for 2 h on 100 V, in 1× TBE buffer. Afterwards, the electrophoresis gel was stained with ethidium bromide and visualized under a UV lamp [17]. The experiments were done twice.

### 2.6. qRT-PCR Assay

Total RNA was extracted from cells using the RNAeasy kit (Qiagen, Venlo, Netherlands), according to the manufacturer’s instructions. Reverse transcription was done with Primesript RTase (Takara, Osaka, Japan) using oligodT. Quantitative real-time PCR (qRT-PCR) was performed using GoTaq^®^ qPCR Master Mix (Promega, Madison, WI, USA) in a Quant Studio cycler (Applied Biosystems, ThermoFisher Scientific, Waltham, MA, USA). Primers for PCR reaction were published previously [18,20] or were designed in Primer-BLAST (https://www.ncbi.nlm.nih.gov/tools/primer-blast/ (accessed on 20 June 2026) NIH, Bethesda, MD, USA). Primers were: HPRT: F: 5′-CTTTGCTGACCTGCTGGATT-3′; R: 5′-TCCCCTGTTGACTGGTCATT-3′; PAI1: F: 5′-CTGGTGAATGCCCTCTACTTC-3′ R: 5′-TGCTGCCGTCTGATTTGT-3′; uPA: F: 5′-GGAGATGAAGTTTGAGGTGGAA-3′, R: 5′-CTCCTTGGAACGGATCTTCAG-3′; uPAR: F: 5′-TTGAAGATCACCAGCCTTACC-3′; R: 5′-GGTAACGGCTTCGGGAATAG-3′; ATF3: F: 5′-CTGCAGAAAGAGTCGGAGAAG-3; R: 5′-CCGATGAAGGTTGAGCATGTA-3′; ATF4: F: 5′-AATGGCTGGCTGTGGATG-3′; R: 5′-TCCAATCTGTCCCGGAGAA-3′; BiP: F: 5′-GGTGCCTACCAAGAAGTCTCA-3′; R: 5′-TGATTGTCTTTTGTCAGGGGTCT-3′; SQSTM1: F: 5′-ACAGGTGAACTCCAGTCCCTA-3′; R: 5′-CTGGGAGAGGGACTCAATCAG-3′; DDIT3 (CHOP): F: 5′-GCAAGAGGTCCTGTCTTCAGAT-3′; R: 5′-GCTTGTGACCTCTGCTGGTT-3′; TRIB: F: 5′-TCAAGCTGTGTCGCTTTGTC-3′; R: 5′-AGCTGAGTATCTCAGGTCCCA-3′; GADD34: F: 5′-GACTGCAAAGGCGGCTCAAG-3′; R: 5′-AGACAGCCAGGAAATGGACAG-3′; GDF15: F: 5′-GGATACTCACGCCAGAAGTG-3′; R: 5′-GAACAGAGCCCGGTGAAG-3′; XBP1: F: 5′-GGAGTTAAGACAGCGCTTGGGGA-3′; R: 5′-TGTTCTGGAGGGGTGACAACTGGG-3′; FOS: F’-AAGGAGAATCCGAAGGGAAAGG-3′; R’-GGCAATCTCGGTCTGCAAAG-3′. Gene expression was validated by comparison with HPRT gene expression, and presented as a relative expression in treated cells in comparison with expression in untreated cells. The experiments were done twice. The results presented stem from one experiment with two technical replicates.

### 2.7. Statistical Analysis

The software package Microsoft Office, Statistica 14.0.0.15 (TIBCO Software, Inc., Palo Alto, CA, USA), and GraphPad Prism 10.5.0 (GraphPad Software, Boston, MA, USA) were used for statistical analysis. The tests used were Student’s *t* test and, for multiple comparison, one-way ANOVA with Tukey post-test. Significance was set at *p*-value < 0.05.

## 3. Results

### 3.1. Modulation of Urokinase Activity After Thapsigargin Treatment

**Figure 1:**
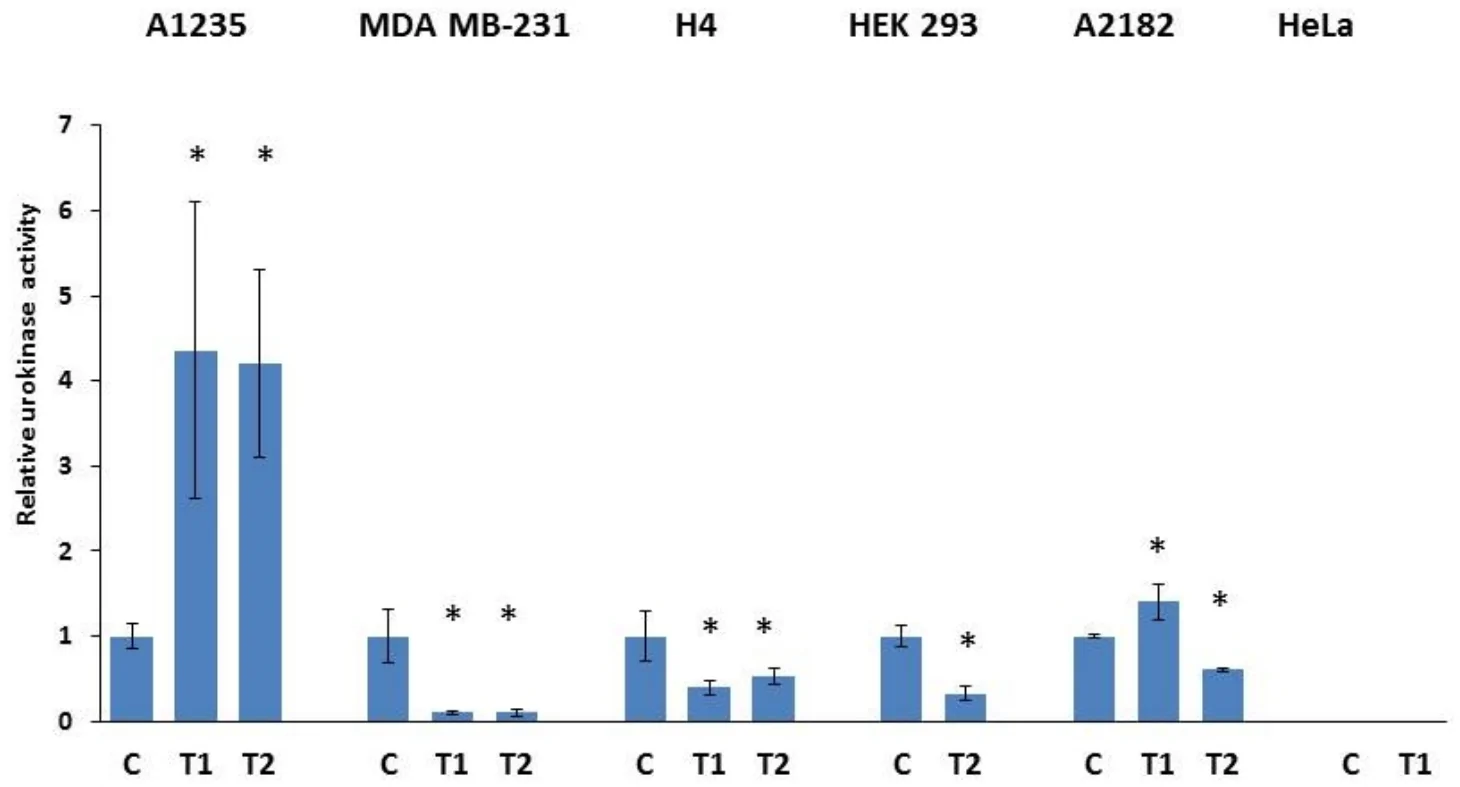
Urokinase activity in different cell lines. Cells were treated with different Thapsigargin concentrations for 24 h. Urokinase activity was determined in conditioned media by radial caseinolysis. Experiments were done in two biological replicates and two enzymatic reactions each, and statistics were done by one-way ANOVA with Tukey post-test. A1235: glioblastoma cell line, MDA MB-231: breast cancer cell line, H4: neuroglioma cell line, HEK 293: human embryonic kidney cells, A2182: lung cancer cell line, HeLa: cervical carcinoma cell line, C: control, T1: Thapsigargin 0.25 µM, T2: Thapsigargin 0.5 µM. * The mean values were significantly different from the control (*p* < 0.05).

Our previous experiments showed that sodium salicylate modulated uPA activity and further investigations established that it could induce ER stress [14,21]. To explore whether ER stress can be responsible for uPA activity regulation, we treated different tumor cell lines with Thapsigargin, a known ER stress inducer acting as a SERCA ATPase inhibitor [13]. After 24 h-treatment with different drug concentrations, uPA activity was determined by caseinolysis in the conditioned media. The results are presented in Figure 1. While upregulation in urokinase activity was found in the conditioned media of A1235 glioblastoma cells, in other cell lines, breast cancer cell line MDA MB-231, neuroglioma H4, human embryonic kidney cell line HEK293, and lung cancer cell line A2182, we detected a decrease in urokinase activity (Figure 1). Some cell lines, such as cervical carcinoma cell line HeLa, did not show urokinase activity and did not change this feature after treatment.

Additionally, urokinase activity modulation by Thapsigargin and sodium salicylate was confirmed in A1235 and MDA MB-231 cells by zymography. These cell lines were chosen as they showed the highest changes in urokinase activity and were previously investigated for response on sodium salicylate [14,22]. Both sodium salicylate and Thapsigargin increased urokinase activity in the glioblastoma cell line, and decreased it in the MDA MB-231 cells (Supplementary Figure S1).

**Figure 2:**
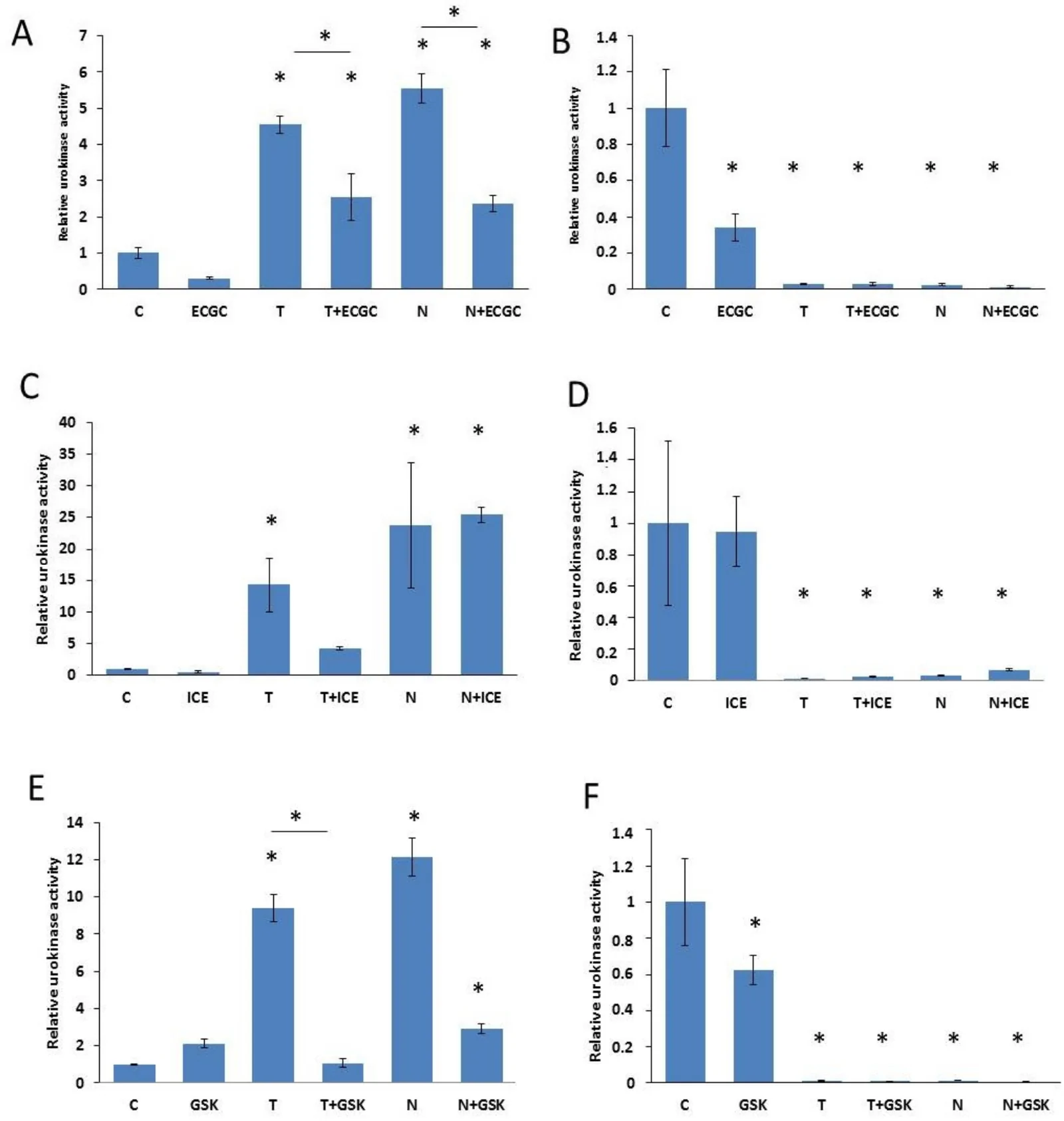
Urokinase activity of A1235 cells (**A**,**C**,**E**) and MDA MB-231 cells (**B**,**D**,**F**) after treatment with Thapsigargin and sodium salicylate in combination with epigallocatechin gallate (**A**,**B**), GSK260614 (**C**,**D**), and Icerguastat (**E**,**F**). Cells were treated with Thapsigargin and sodium salicylate for 24 h. Urokinase activity was determined by caseinolysis of collected conditioned media. Experiments were done in two biological and two enzymatic reactions for each. Statistical analysis was done by one-way ANOVA with Tukey post-test. ECGC: 100 µM epigallocatechin gallate; GSK: 10 µM GSK260614, ICE: 10 µM Icerguastat, T: Thapsigargin, 0.5 µM for A1235 cells and 0.25 µM for MDA MB-231 cells, N: 15 mM sodium salicylate. * The mean values were significantly different from the control (*p* < 0.05).

To further investigate the role of ER stress in uPA activity modulation, we treated A1235 and MDA MB-231 cells with sodium salicylate and Thapsigargin in combination with UPR molecules’ inhibitors. Cells were treated with epigallocatechin gallate ECGC (used as BiP inhibitor), Icerguastat (used as IRE1α inhibitor) and PERK inhibitor GSK2606414. The results (Figure 2) showed that ECGC alone inhibited basal uPA activity. ECGC also inhibited Thapsigargin and NaS induced uPA activity in A1235 cells (Figure 2A). In MDA MB-231 cells, uPA inhibition caused by Thapsigargin and NaS was not significantly changed after ECGC treatment (Figure 2B). IRE1α inhibitor Icerguastat in glioblastoma cells decreased uPA activity induction by Thapsigargin, but did not significantly change NaS-induced uPA activity (Figure 2C). In MDA MB-231 cells there was no change in basal uPA activity, nor did Thapsigargin and NaS inhibition change after Icerguastat treatment (Figure 2D). PERK inhibitor did not influence basal urokinase activity in A1235 cell, while in MDA MB-231 cells it was decreased. In the glioblastoma cell line, PERK inhibition decreased urokinase activity upraised by drug treatment, while in MDA MB-231 cells, inhibition remained even after combined treatment (Figure 2E,F).

### 3.2. uPA System Molecules Expression After ER Stress

**Figure 3:**
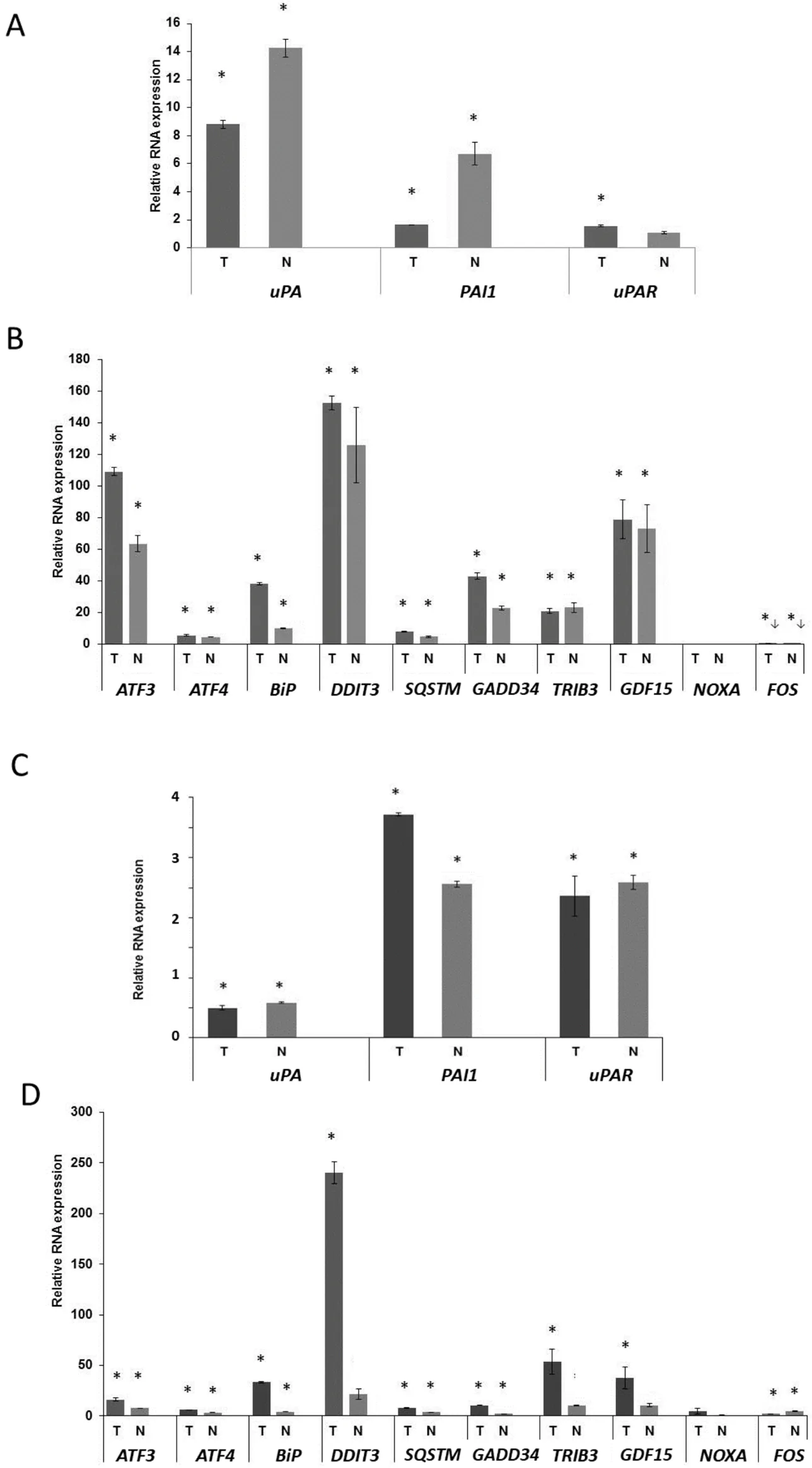
RNA expression analysis of uPA system (**A**,**C**) and ER stress related genes (**B**,**D**) in A1235 (**A**,**B**) and MDA MB-231 (**C**,**D**) cells after treatment with Thapsigargin and sodium salicylate. Cells were treated with 0.5 µM Thapsigargin or 15 mM sodium salicylate for 24 h, when they were collected and RNA isolated. After reverse transcription samples were analyzed by qRT-PCR. Results are presented as fold induction in comparison with control. The control gene expression analyzed was *HPRT*. Samples were analyzed in technical duplicates. Statistical analysis was done by one-way ANOVA with Tukey post-test. T: samples from cells treated with Thapsigargin, N: samples from cells treated with sodium salicylate. Names of genes analyzed are on x axis. ↓: downregulation * The mean values were significantly different from the control (*p* < 0.05).

**Figure 4:**
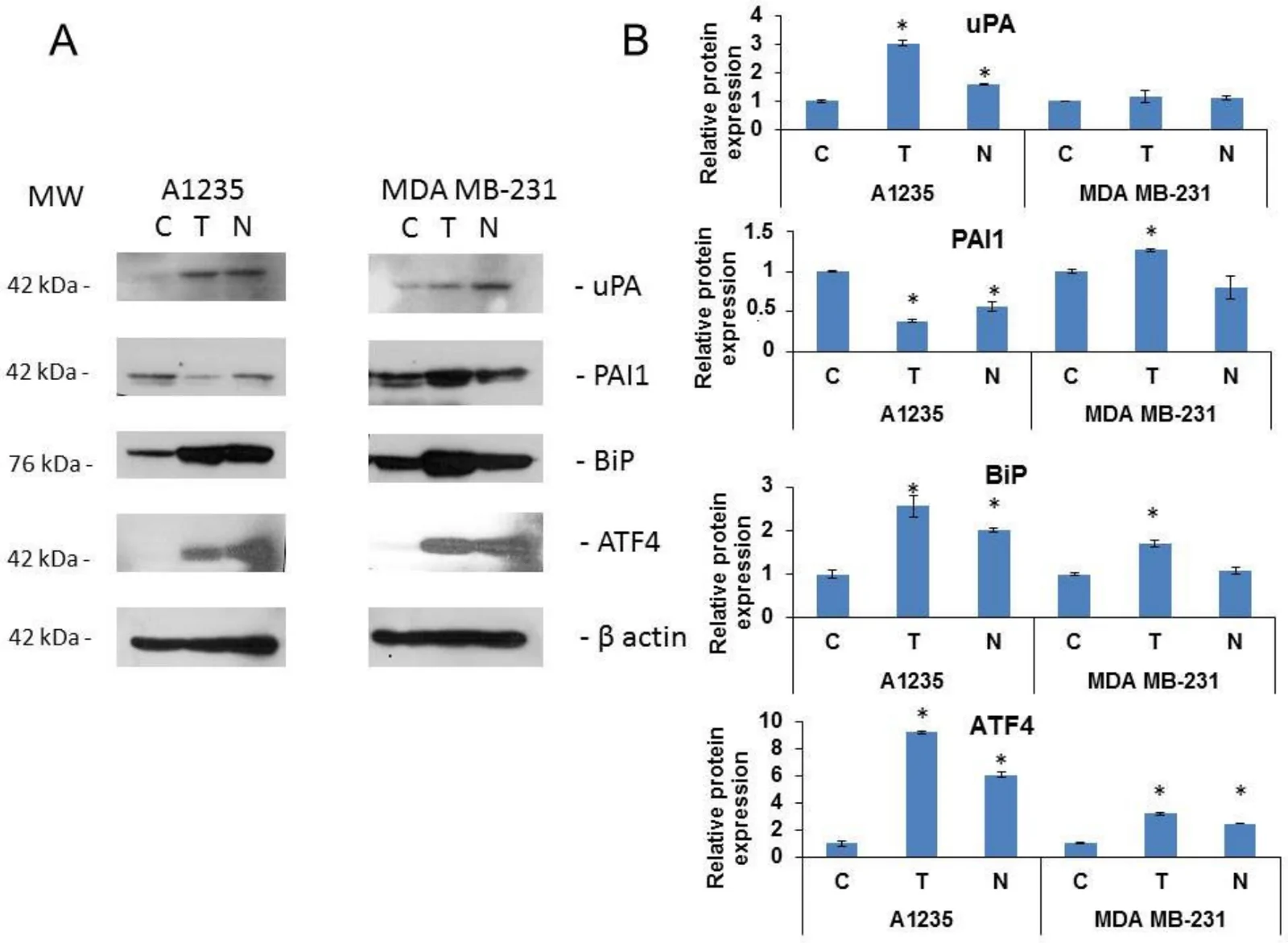
Western blot analysis of uPA system and ER stress related proteins in A1235 and MDA MB-231 cells after treatment with Thapsigargin and sodium salicylate. Cells were treated with 0.5 µM Thapsigargin or 15 mM sodium salicylate for 24 h, when protein lysates were prepared and analyzed by Western blot. Proteins were detected by chemiluminiscence on X ray films (**A**), and analyzed by densitometry (**B**). Experiments were done twice and representative blots are presented. Analysis was done by Student’s T test. T: samples from cells treated with Thapsigargin, N: samples from cells treated with sodium salicylate. * The mean values were significantly different from the control (*p* < 0.05).

uPA activity depends on expression of both uPA and PAI1 and their balance. Therefore, we analyzed the expression of urokinase and its inhibitor at the protein and RNA level. Preliminary RT-qPCR results showed that in the A1235 cells, after both Thapsigargin and NaS treatment, *uPA* expression was increased. *PAI1* was increased after NaS treatment, and *uPAR* after thapsigargin treatment (Figure 3A). Changes in the uPA:PAI1 ratio were also observed at the protein level. After Thapsigargin treatment, expression of uPA was increased, and PAI1 decreased. Similarly, after cell treatment with NaS, uPA expression was increased and PAI1 decreased, in comparison with untreated cells (Figure 4). When MDA MB-231 cells were treated with Thapsigargin, *uPA* expression was 50% of the basal level, while *PAI1* and *uPAR* expression was increased. Treatment with NaS also decreased *uPA* expression by ~50% and increased *PAI1* expression (Figure 3C). On the protein level, uPA expression was similar in control and Thapsigargin and sodium salicylate treated cells. However, increase in PAI1 expression was detected after Thapsigargin treatment (Figure 4).

### 3.3. Expression of Unfolded Protein Response Molecules After ER Stress

Expression of molecules involved in the unfolded protein response was analyzed in A1235 and MDA MB-231 cells after treatment with ER stress inducer and sodium salicylate. We analyzed the expression of *ATF4*, a UPR downstream transcription factor known for its expression in ER and integrated stress response, *ATF3*, a transcription factor in stress response, BiP, an ER chaperone, *DDIT3* or *CHOP*, a transcription factor in ER stress response, *TRIBB3*, a regulator in integrated stress response, and Sequestosome 1 (*SQSTM1/p62*), a protein involved in a protein destruction pathways [23,24]. Expression of *GDF15*, *NOXA,* and *FOS* was also analyzed. GDF15 is a TGFβ superfamily member, induced by sodium salicylate, and FOS is a transcription factor activated by the MAPK pathway involved in the stress response [25]. NOXA is a Bcl2 family member involved in apoptosis induction [26]. In A1235 cells, Thapsigargin increased expression of all ER stress related genes analyzed, namely *ATF4*, *ATF3*, *BiP*, *TRIBB3*, *SQSTM1,* and *DDIT3*. The expression of the same genes was also increased after cell treatment with sodium salicylate (Figure 3B). *NOXA* was not expressed in glioblastoma cells, while *FOS* showed decreased expression after NaS and Thapsigargin treatment. On the protein level, both, Thapsigargin and sodium salicylate induced expression of BiP and ATF4 in A1235 cells (Figure 4).

In MDA MB-231 cells, most of ER stress related genes analyzed had an increased expression after NaS and Thapsigargin treatment. Treatment with NaS induced ER stress gene expression to a lower level in comparison with Thapsigargin. *NOXA* was not significantly induced by Thapsigargin and NaS, but was expressed. *FOS* expression was increased after Thapsigargin and NaS treatment. At the protein level, Thapsigargin induced only BiP, and both Thapsigargin and sodium salicylate induced ATF4 (Figure 3D and Figure 4).

**Figure 5:**
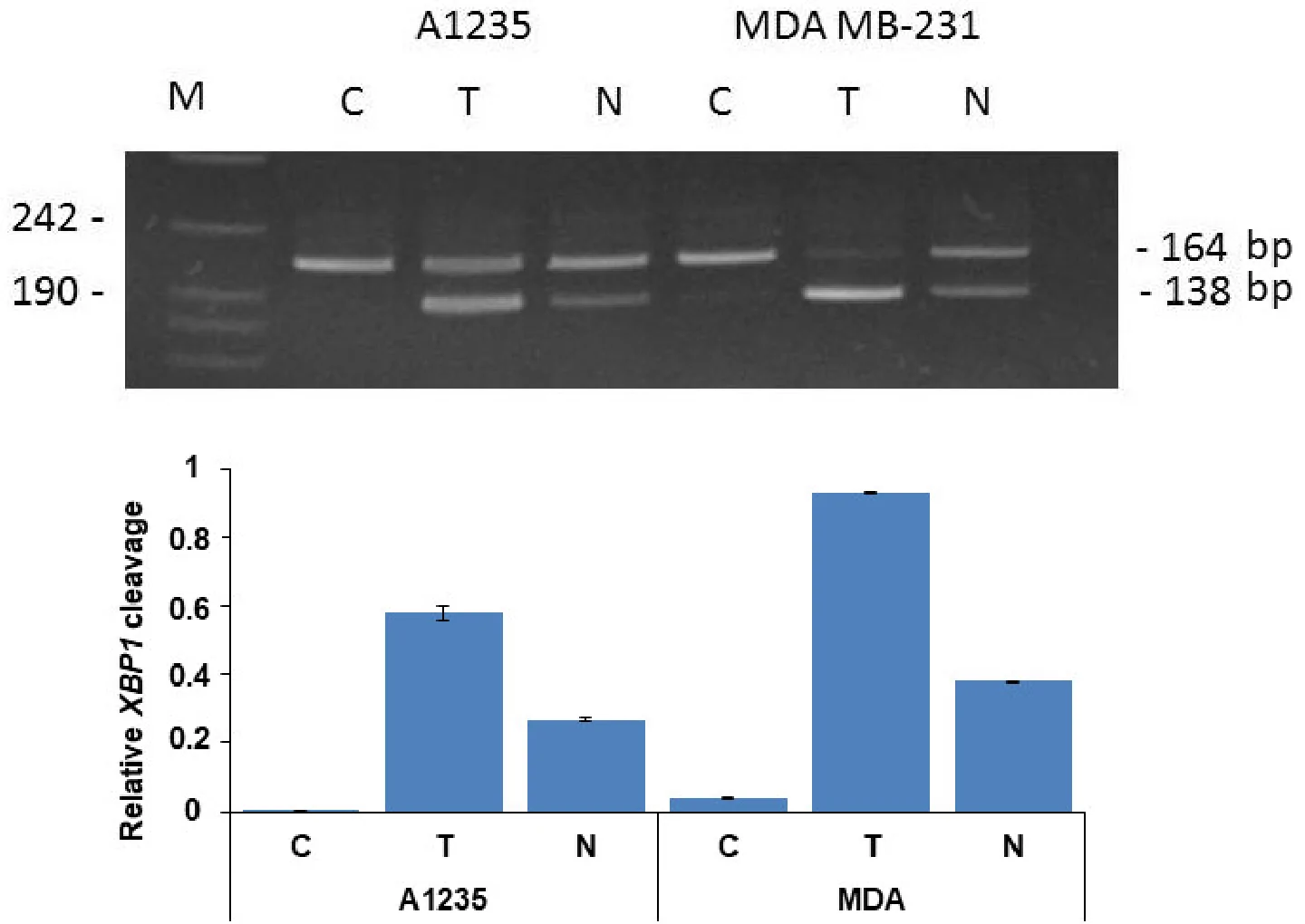
PCR analysis of XBP1 cleavage by IRE1α activation in A1235 and MDA MB-231 cells, after treatment with 0.5 µM Thapsigargin and 15 mM sodium salicylate. At 24 h after cell treatment, RNA was isolated from the cells, cDNA was produced, and PCR analysis was done with *XBP1* primers. Amplicons were run on polyacrylamide gel and relative *XBP1* cleavage was determined as a ratio between small sequence amount (138 bp) and total amount (164 bp + 138 bp). Amplicon amounts were determined by densitometry. M: DNA standard, C: control, T: Thapsigargin, N: sodium salicylate, MDA: MDA MB-231 cells.

As most of the analyzed molecules belong to the PERK pathway, we also analyzed the activation of the IREα1 pathway by *XBP1* mRNA cleavage. The sample cDNA was amplified with common primers which produced sequences of different length due to mRNA cleavage [20]. Activation of the IREα1 pathway was detected in both cell lines after treatment with NaS and Thapsigargin, but the most prominent activation was present after treatment with Thapsigargin (Figure 5).

### 3.4. Effect of ER Stress on Cell Migration, Invasion and Proliferation

**Figure 6:**
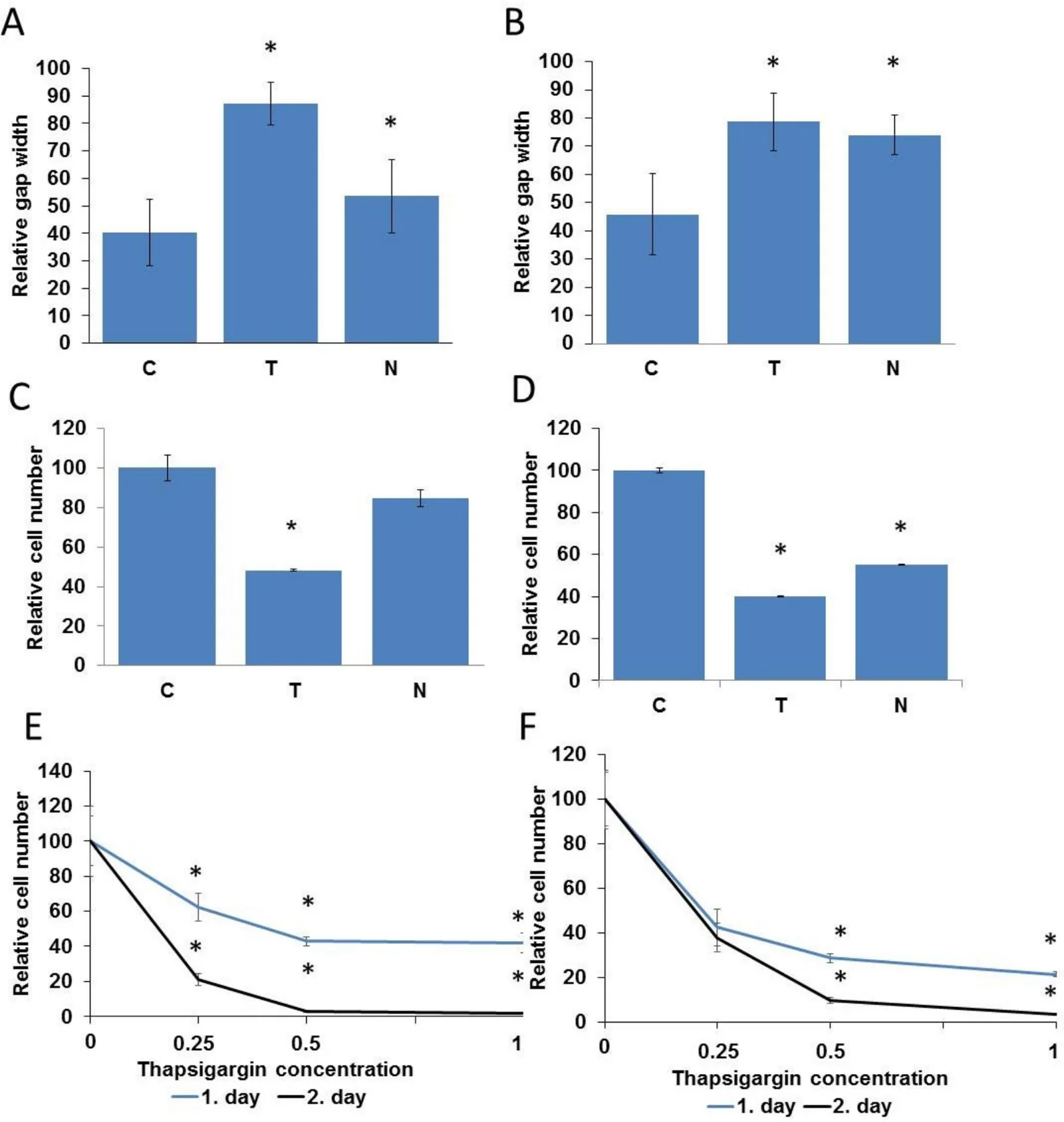
Analysis of migration (**A**,**B**), invasion (**C**,**D**), and proliferation (**E**,**F**) of A1235 (**A**,**C**,**E**) and MDA MB-231 cells (**B**,**D**,**F**) treated with Thapsigargin and sodium salicylate. Cell migration was analyzed by scratch assay after ~20 h. Gap width was measured on microphotographs taken immediately after cell layer scratching and after ~20 h. Two biological samples per treatment and at least nine points per sample were analyzed. Cell invasion was analyzed by migration through the transwell membrane covered with extracellular matrix. The cell number was determined by counting stained cells. Two biological samples per treatment were analyzed. Cell proliferation was determined by counting cells in triplicate after one and two days of treatment with different Thapsigargin concentrations. Statistical analysis was done by one-way ANOVA with Tukey post-test. C: control cells, T: cells treated with Thapsigargin, 0.5 µM for A1235 cells and 0.25 µM for MDA MB-231 cells, N: 15 mM sodium salicylate. * The mean values were significantly different from the control (*p* < 0.05).

The urokinase system and its molecules are involved in the regulation of cell adhesion, migration, and invasion [1,27]. Therefore, we examined the influence of ER stress on cell migration and invasion. When treated with Thapsigargin and NaS, glioblastoma A1235 cells showed decrease in cell migration, despite increase in the urokinase activity. Similarly, MDA MB-231 cells had decreased migration after treatment (Figure 6A,B). When cells had to migrate through the extracellular matrix after Thapsigargin treatment in the cell invasion test, both cell lines showed decreased rate of invasion (Figure 6C,D).

We also analyzed cell proliferation after treatment with Thapsigargin. A1235 and MDA MB-231 cells were previously tested for NaS effects on cell proliferation, which was inhibited in both cell lines [14,22]. Thapsigargin also influenced cell growth. Treatment with different concentrations of Thapsigargin showed that more than 40% of A1235 cells survived at 1 µM concentration after 24 h, but less than 10% after 48 h. MDA MB-231 cells were more sensitive to Thapsigargin, with less than 30% survival after 24 h treatment with 1 µM concentration, but higher survival after 48 h in comparison with glioblastoma cells. A1235 cells had IC_50_ 0.41 µM Thapsigargin after 24 h, but 0.16 µM after two days. MDA MB-231 cells had similar IC_50_ values on the first and second day, 0.197 µM and 0.22 µM, respectively (Figure 6C).

## 4. Discussion

ER stress response is a mechanism of cell and organism defense from accumulated unfolded or misfolded proteins, aiming to establish homeostasis or to direct cells to apoptosis. The process increases expression of chaperones, initiates changes in lipid metabolism, downregulation of protein translation and, depending on the degree of the ER stress, causes autophagy and apoptosis [28]. Translational arrest is dependent on the proteins/RNA sequences involved, so the ratio of different proteins can be changed, including extracellular proteins. However, our hypothesis that ER stress modulates urokinase activity came from experimental data: while our previous experiments showed that both A1235 and MDA MB-231 cells change urokinase activity after treatment with sodium salicylate [14,22], Silva et al. and Genz et al. showed that sodium salicylate can cause ER stress response [21,29]. Therefore, we analyzed whether Thapsigargin, an ATPase SERCA inhibitor, commonly used as ER stress inducer, can modulate urokinase activity, and how different cell lines respond to ER stress.

The results showed that Thapsigargin and sodium salicylate induced ER stress response in both the glioblastoma and breast cancer cell line. This was shown by changes in expression of unfolded protein response-related molecules, particularly PERK-pathway related genes, such as *ATF4* and *ATF3*, *TRIBB3*, *GADD34*, *CHOP/DDIT3,* and chaperone *BiP*. Expression of ATF4 and BiP was also mirrored in protein expression. Additionally, activation of IREα1 pathway was shown by *XBP1* activation. ATF6 pathway was not analyzed, but it could also influence chaperone and *XBP1* expression and activity, and it was found to be involved in the adaptation to long-term chronic stress [30]. Expression of UPR genes after Thapsigargin, Tunicamycin, and numerous other ER-stress inducible agents in different cell lines has been shown [31,32,33]. A complex regulation through mutual induction and feedback loops of the main molecules in the ER stress response, such as TRIBB3, GADD34, DDIT3, and ATF4 has been revealed and is responsible for the fate of the cell [24,34,35]. ERS gene induction after Thapsigargin was higher in glioblastoma cells than in MDA MB-231 cells in *ATF3*, *GADD34,* and *TRIBB3* expression, while other genes had similar fold induction. High basal expression of some UPR genes, such as BiP, and robust response to stress could explain the high resistance of A1235 cells to Thapsigargin and possibly other chemotherapeutics. Modulation of ATF4 can increase cancer cell survival under stress [35]. Lorenz et al. [36] found that ATF4 mediates the adaptation of human glioblastoma cells to hypoxia and alkylating agents, and increase their viability. Induction of UPR increased chemoresistance and changed metabolism in glioma cells [37]. ATF3 was found to be a crucial stress responsive gene of glia and neurons and was found to be conserved pro-regenerative factor [38,39].

Considering cell proliferation, many chemotherapeutics inhibit cell growth and proliferation, through cell cycle arrest induced by cell and ER stress, in addition to the possibility of their cytotoxicity [40]. Thapsigargin and NaS increased growth inhibition in correlation with their concentration [14,22]. Beside the direct influence of ER stress on cell proliferation, the uPA–uPAR system can also both induce and inhibit cell proliferation, depending on the cell microenvironment and cell-specific signaling [41,42,43]. A1235 cells appear relatively resistant to Thapsigargin, with IC_50_ around 0.4 µM after 24 h of treatment. Possibly high basal and induced levels of UPR genes and proteins enable them for short-term survival, in comparison with other cell lines. Prolonged exposure to ER stress led to higher cytotoxicity in A1235 cells, in comparison with MDA MB-231: less then 5% A1235 cells survived, in comparison with 10% of MDA MB-231 in conditions of 0.5 µM Thapsigargin treatment, and IC_50_ was lower than in MDA MB-231 cells. In a glioblastoma cell line, *NOXA* expression was very low or missing, possibly leading to resistance on apoptosis. At the same time, *NOXA* was increased in MDA MB-231 cells, and Cano-Gonzalez et al. [26] showed the involvement of caspase 8 and NOXA in apoptosis caused by ER stress in triple negative breast cancer cells.

Changes in cell migration, invasion and proliferation can be considered to be a direct cell response to ER stress. Urokinase activates plasminogen, and plasmin can degrade the extracellular matrix and thus potentially increase invasion. Also, the interplay among uPA, uPAR, and PAI1 and extracellular matrix proteins, such as vitronectin, uPAR cofactors, and other molecules, can regulate cell adhesion, migration, and proliferation [27,44,45]. The uPA system can also increase metalloprotease activation, which is directly involved in extracellular degradation and invasion [46]. However, in both cell lines, inhibition of migration and invasion was detected, despite the increase of urokinase activity in A1235 cells. Although treated cells showed inhibition of proliferation, the conditions of treatment were sublethal (Figure 6E,F). The uPA system is not the only mechanism involved in migration and invasion. It is possible that the inhibition of other involved pathways and translational arrest dominated over urokinase activity in regulating A1235 cell invasion, from integrin activation in adhesion to signal transduction and cytoskeletal regulation. In experiments where retinoic acid treatment of A1235 cells increased urokinase activity, cells also had decreased migration, but unchanged proliferation. In these cells, retinoic acid and sodium salicylate inhibited MMP2 expression [18] [preliminary RNA-seq data]. Also, the expression of integrins was changed: retinoic acid decreased the expression of β5 and αv integrins [18]. Treatment with sodium salicylate could potentially also decrease expression of some integrin subunits important for glioblastoma metastasis such as αv [47] and [according to preliminary RNA-seq data]. Cell migration can also be inhibited due to strong protein synthesis inhibition and stress conditions. Therefore, inhibition of migration seems to be caused by generalized cellular stress, rather than changes in the uPA system activity. However, some scientists found that moderate PERK activation increased cell migration and invasion in medulloblastoma through VEGFA signaling, and in colorectal cancer through the FOXM1 pathway [48,49]. In addition, low Thapsigargin treatment was found to increase invasion through ER stress-induced expression of various long noncoding RNA in different types of cancer [50].

Urokinase regulation after ER stress displayed cell-type specificity. Most of the cell lines decreased uPA activity and their Thapsigargin resistance was dependent on the cell line. A similar response has been already shown in sodium salicylate [22]. The only exceptions were A1235 cells with increased urokinase activity after both treatments. These cells showed an increase in urokinase activity after treatment with some other chemotherapeutics and DNA damaging agents [51]. At the RNA level, an increase in both *uPA* and *PAI1* was detected after NaS and Thapsigargin treatment, although the *uPA* increase was much higher. We have already shown activation of the *uPA* promoter after NaS treatment in glioblastoma cells [14]. At the protein level, besides an increase in uPA, a decrease in PAI1 expression was detected. Final activity depends on the ratio of extracellular uPA and PAI1. Their coregulation has been also previously shown [22,52,53]. Changes in PAI1 expression may be due to post-transcriptional or post-translational regulation, i.e., to the activity of several miRNA [54,55,56]. In MDA MB-231 cells, uPA activity after ER stress was decreased. This response seems to be characteristic for most cell lines, and it was similar to the response for NaS [22]. uPA activity downregulation can be explained by a decrease in *uPA* transcription and an increase in *PAI1* mRNA. At the protein level, an increase in the PAI1 level after Thapsigargin treatment was detected. In previous experiments we also detected changes in the uPA:PAI1 ratio [53]. Besides transcription, the ratio can be changed as a consequence of different rates of translation. In addition, PAI1 was shown to be increased in the recovery time of ER stress in the human liver cell line, as well as under conditions of cell stress [57,58]. It was also shown that senescence suppresses the integrated stress response and can induce the cell secretory phenotype, which can include PAI1 secretion [59,60]. All these data indicate several levels of uPA system regulation, from transcription to translation and post-translational regulation, and indicate the fine tuning of uPA and PAI1 expression through common signaling pathways.

Experiments with ER stress inhibitors showed that inhibitors alone can modulate basal uPA activity. ECGC is considered to be a BiP inhibitor, but it also has different intracellular targets, and was shown to regulate urokinase expression through regulation of its promoter and degradation of uPA mRNA, and through inhibition of ERK and p38 pathways and other signaling pathways [61,62]. Consequently, ECGC treatment inhibited basal uPA activity and also decreased uPA activity induction by Thapsigargin and NaS. Icerguastat, used as a IREα1 inhibitor, decreased Thapsigargin-induced uPA activity in A1235 cells, whereas no significant effect was observed in MDA-MB-231 cells. GSK2606414, used as a PERK inhibitor, decreased urokinase activity, which was already modulated by ER stress inducers in glioblastoma cells. This response could indicate the involvement of PERK pathways in urokinase regulation. It should be also mentioned that urokinase activity induction after treatment of A1235 cells with different concentrations of chemotherapeutics showed a bell-shaped curve with maximal response at optimal treatment concentrations [53]. ER stress inducers could also show such response [14]. Therefore, additional stress caused by PERK inhibitor treatment could lead to decrease in urokinase activity. PERK signaling pathways also involve feed-back loops, which could additionally influence the outcome of its pathway inhibitors [24,35].

To exclude possible effects of calcium intracellular increase caused by Thapsigargin treatment, we also did experiments with ionomycin, a potent ionophore, which could increase intracellular calcium level (personal observation). This treatment of both cell lines did not significantly influence either urokinase activity or cell viability. In addition, while Thapsigargin induces ERS through calcium deregulation, sodium salicylate could possibly directly cause ER stress, but also initiate UPR response through cellular stress and by influencing different cellular signaling pathways. It is known to modulate NF-κB and MAP kinase signaling, as well as AMPK, and these responses were found to be cell specific [63,64,65,66,67]. These data could explain the different responses of MDA MB-231 cells on Thapsigargin and NaS.

Although we did not show a direct link between the transcriptional control of the urokinase system with ER stress proteins, we can hypothesize that specific urokinase regulation in A1235 cells could be linked to the binding of different combinations of AP1 transcription factor dimers to the urokinase promoter, as its main regulators. ATF factors can be one of the dimerization members [2]. High expression of *ATF* family transcription factors, both at the basal level and after ER stress, could possibly regulate the uPA promoter. Another AP1 member is FOS, whose RNA expression was found to be different in A1235 and MDA MB-231 cells. It was increased in MDA MB-231 cells, as in other cell lines under ER stress [31], while it had low expression and was decreased in A1235 cells. It can be involved in both, *uPA,* and *PAI1* promoter regulation [68,69]. One of the AP1 members is also JUND, which was found to have cell-specific regulation. It regulates a set of migration-dependent genes, among which is urokinase. In some cell types JUND oppositely regulated uPA system genes, and was found to both decrease and increase cell migration, depending on the cell line-specific signaling and the different affinity of AP1 members toward promoter binding [70]. In addition, several aggressive breast cancer lines have overexpression of Fra-1, a FOS family member of AP1, which can also regulate transcription cells specifically [68].

Chemotherapeutics increase ER stress in cancer cells, which already have a higher stress burden, and can lead to their death. At the same time, cancer cells can have increased resistance to ER stress, due to overexpression of UPR genes, and decreased sensitivity on therapeutic agents. This investigation showed that, besides the direct effects on the tumor cells and their migration and invasion capabilities, ER stress could influence the cell microenvironment and blood coagulation balance in the organism. Therefore, further investigation of cellular-specific responses and chemicals able to modify ER stress response are needed.

## 5. Conclusions

In conclusion, ER stress was found to induce changes in urokinase activity in two cell lines; in a glioblastoma cell line, activity was increased, and in breast cancer cells it was decreased. Modulation was caused by changes in urokinase and PAI1 expression and their ratio. Different regulation of the uPA system genes could be influenced by the different activity of signaling pathways, which is deregulated in cancer cells. Specific signaling pathways responsible for urokinase activity modulation need further investigation. Nevertheless, ER stress caused cell proliferation inhibition in both cell lines, as well as a decrease in cell migration and invasion.

## Data Availability

The original contributions presented in this study are included in the article/Supplementary Material. Further inquiries can be directed to the corresponding author.

## Supplementary Materials

The following supporting information can be downloaded at: https://www.mdpi.com/article/10.3390/biom16091235/s1. Figure S1: Urokinase activity in A1235 glioblastoma cell line and MDA MB-231, breast cancer cell line. Original Western blot images are provided in the Supplementary Materials.
