# Supplementary material for: Influence of Endoplasmic Reticulum Stress on Urokinase Plasminogen Activation
Source: Biomolecules. 2026 Aug 26;16(9):1235. doi: 10.3390/biom16091235 (PMC13604286; doi:10.3390/biom16091235)
Supplement: Supplementary file 1 [file biomolecules-16-01235-s001.zip › biomolecules-4431850-figure S1.pdf]

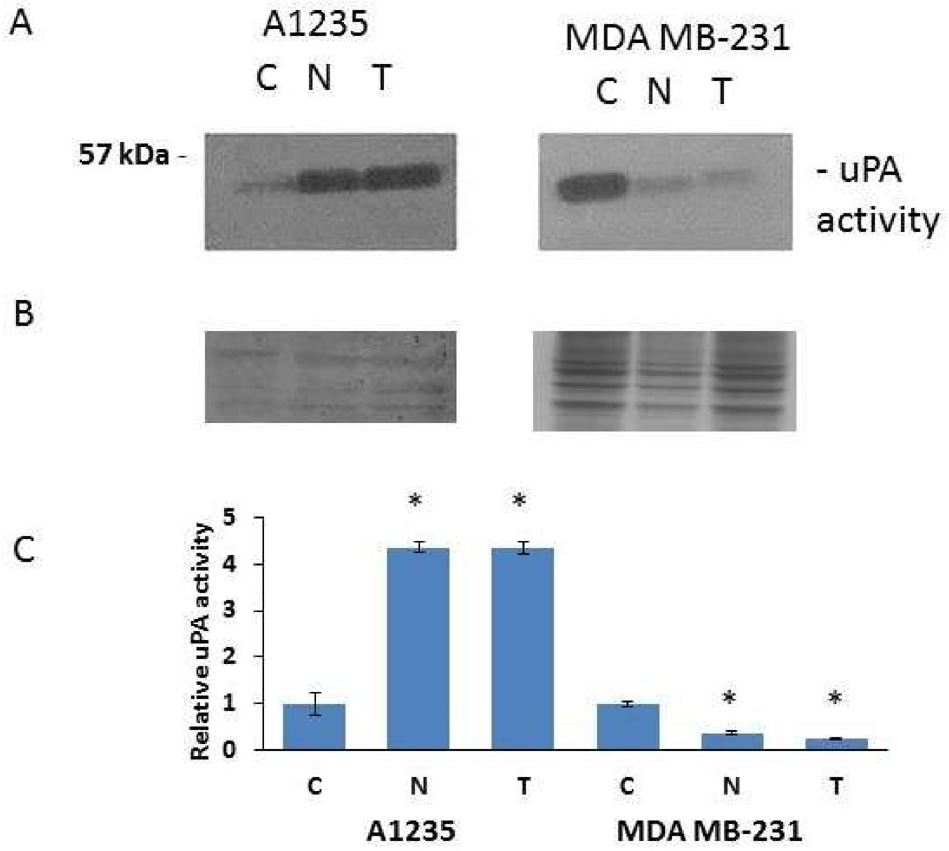

Figure S1. Urokinase activity in A1235 glioblastoma cell line and MDA MB-231, breast cancer cell line. Urokinase activity was determined by zymography of conditioned media (A). Cells were treated with Thapsigargin and sodium salicylate for 24 h. Conditioned media were collected, concentrated and run on polyacrylamide gel. Gel was laid over a substrate gel containing plasminogen and casein. Total cell proteins obtained by cell lysis were run on polyacrylamide gel and stained with Coomasie blue (for MDA MB-231 cells), or transferred to the membrane which was then stained with Amido Black (for A1235 cells) (B). Lysis zones produced by urokinase and bands obtained from total cell proteins were scanned and analyzed by densitometry (C). C: control cells medium, N: medium from cells treated with 15 mM sodium salicylate, T:medium from cells treated with Thapsigargin, 0.5 μM for A1235 cells and 0.25 μM for MDA MB-231. Results were analyzed by Student T test. *The mean values were significantly different from the control ($p < 0.05$).
