# Supplementary material for: Influence of Endoplasmic Reticulum Stress on Urokinase Plasminogen Activation
Source: Biomolecules. 2026 Aug 26;16(9):1235. doi: 10.3390/biom16091235 (PMC13604286; doi:10.3390/biom16091235)
Supplement: Supplementary file 1 [file biomolecules-16-01235-s001.zip › Original Western blots.pdf]

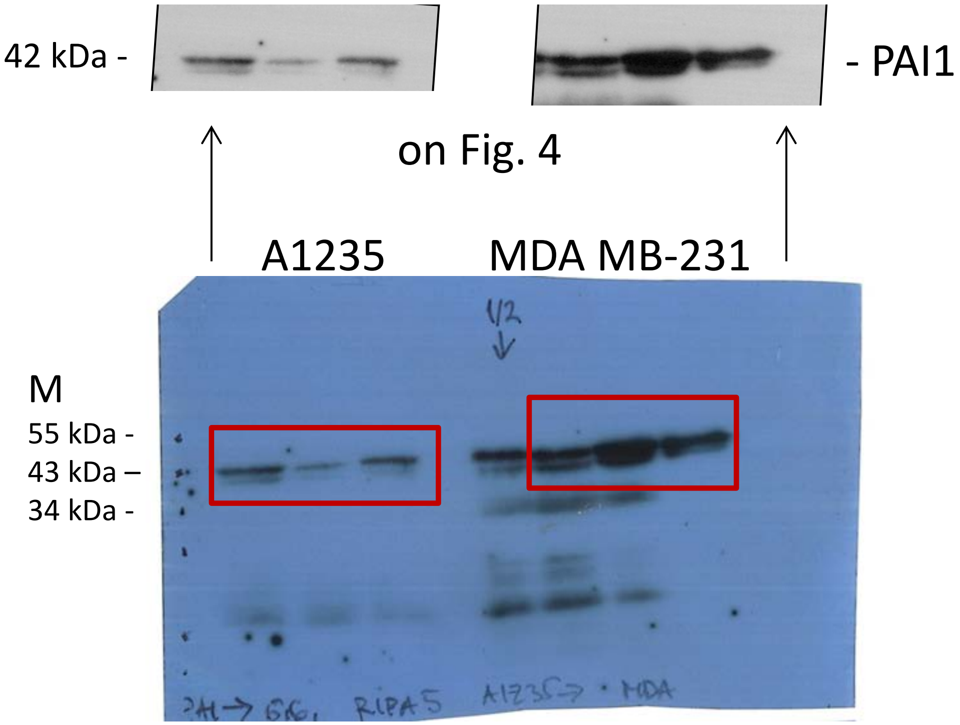

PAI1 detection

Protein marker: Blue Prestained Protein Marker Broad Range 11-250, CST 59329
Marker was not visable on the film, but was labeled after superimposing on the membrane.

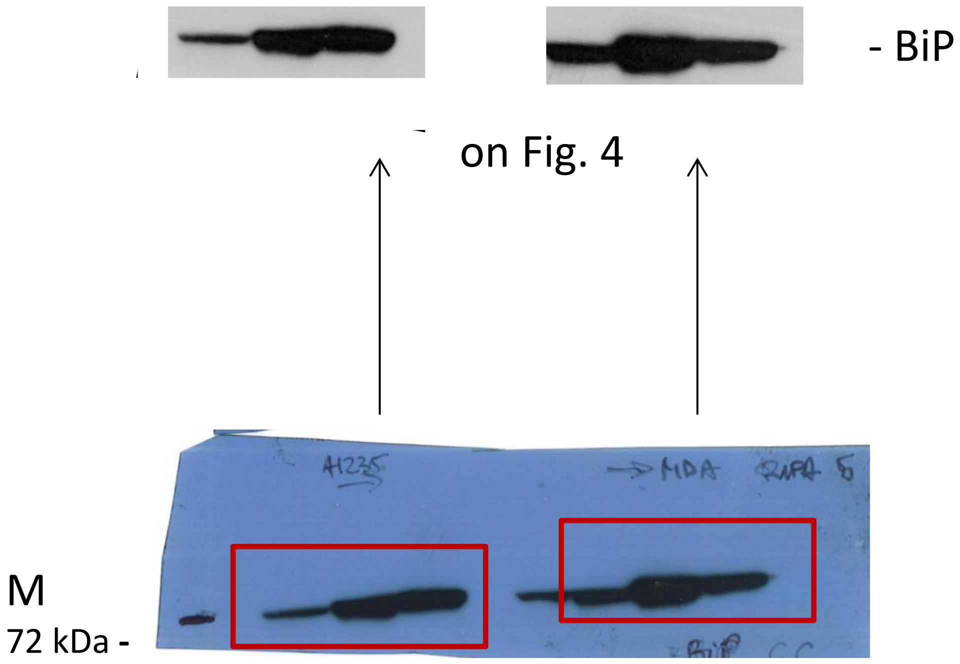

BiP detection

Protein marker: Blue Prestained Protein Marker Broad Range 11-250, CST 59329
Marker was not visable on the film, but was labeled after superimposing on the membrane.

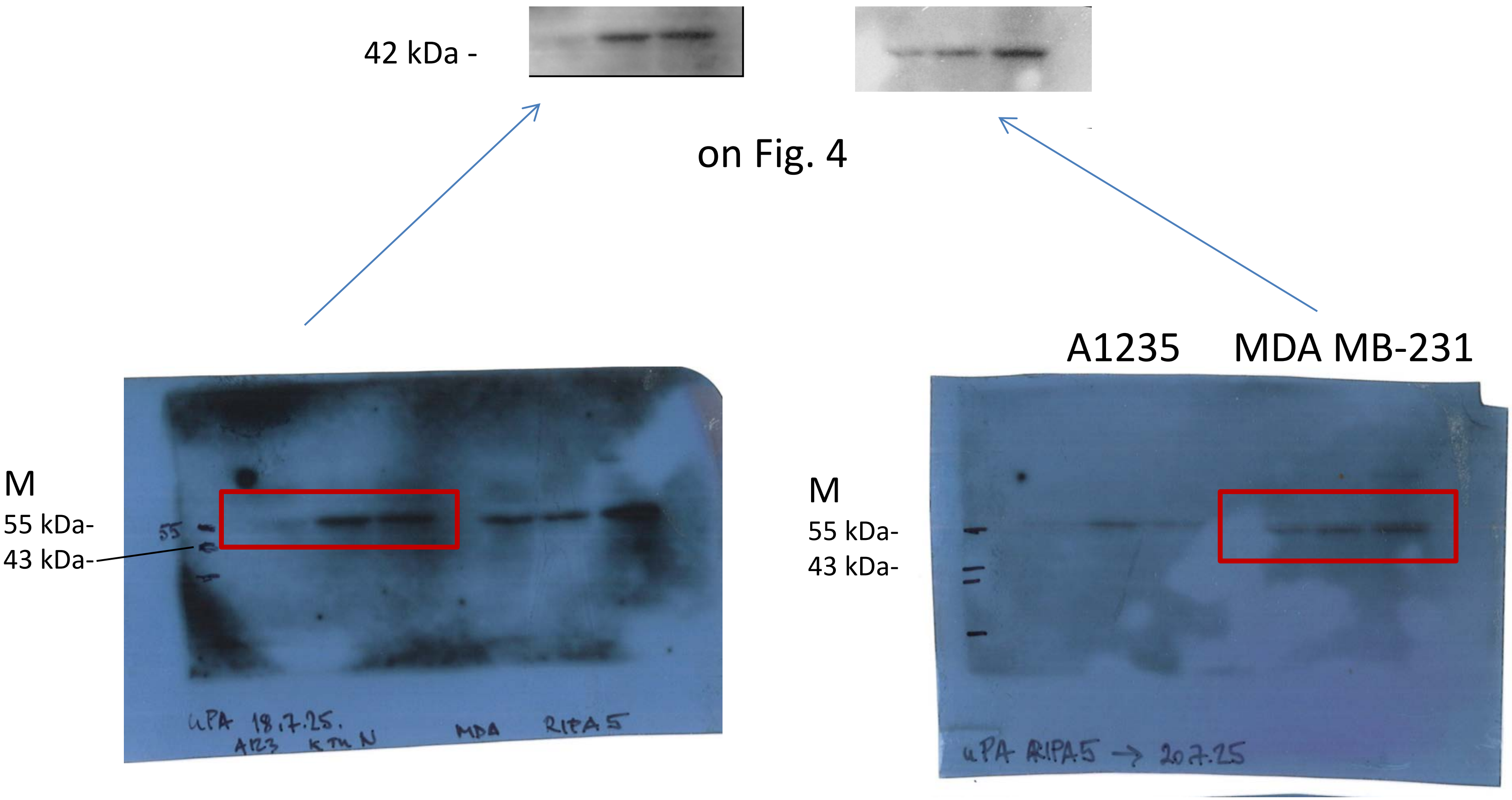

uPA detection

Protein marker: Blue Prestained Protein Marker Broad Range 11-250, CST 59329
Marker was not visable on the film, but was labeled after superimposing on the membrane.

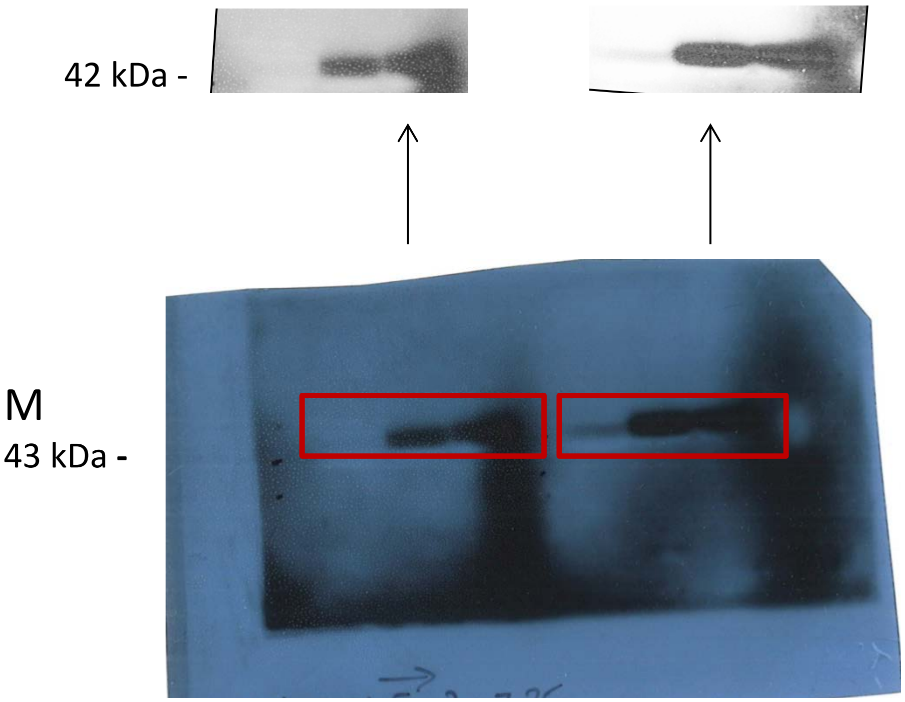

ATF 4 detection

Protein marker: Blue Prestained Protein Marker Broad Range 11-250, CST 59329
Marker was not visable on the film, but was labeled after superimposing on the membrane.

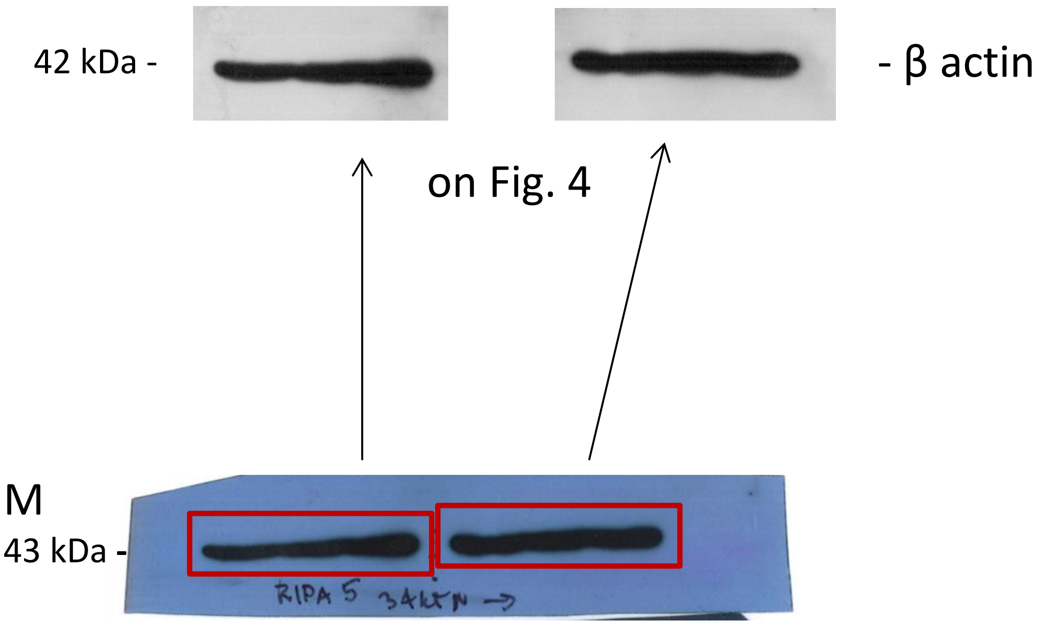

β actin detection

Protein marker: Blue Prestained Protein Marker Broad Range 11-250, CST 59329
Marker was not visable on the film, but was labeled after superimposing on the membrane.

Zymography of cell conditioned media for urokinase activity

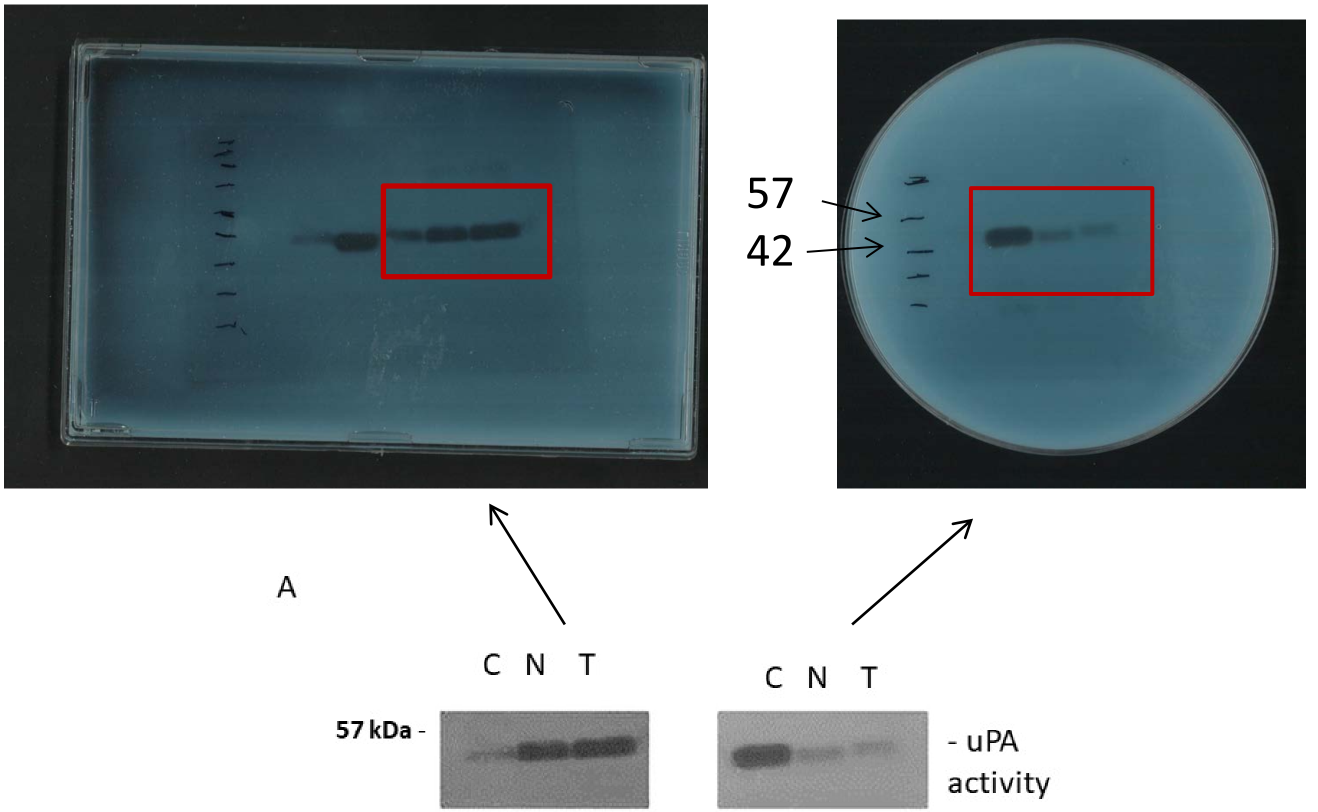

agarose/casein/plasminogen gel plates were overlaid with PAA gel after electrophoresis and lysis zones are visible;
markers were signed according to the position on the gel (which was removed; Blue Prestained Protein Marker Broad Range 11-250, CST 59329)

M

DNA Marker
VIII Roche

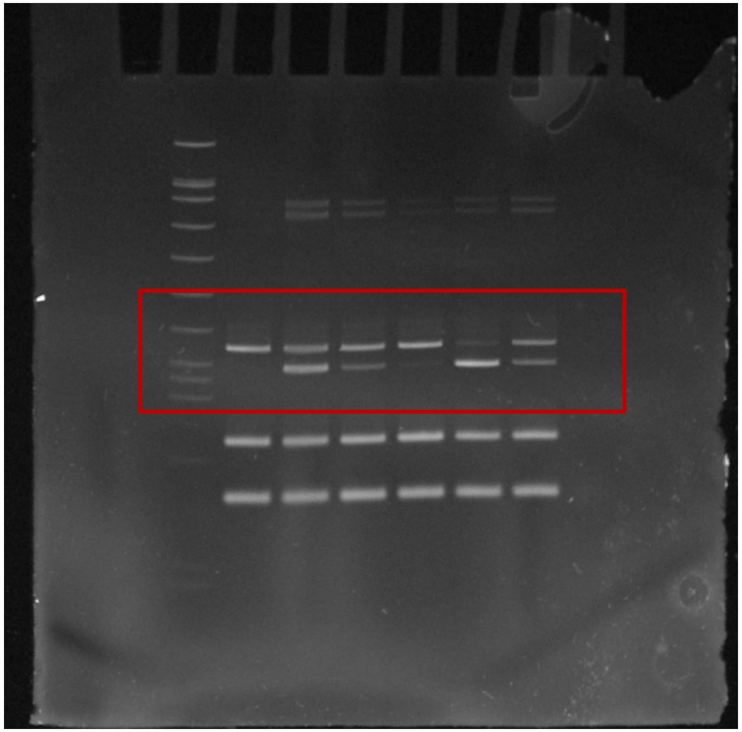

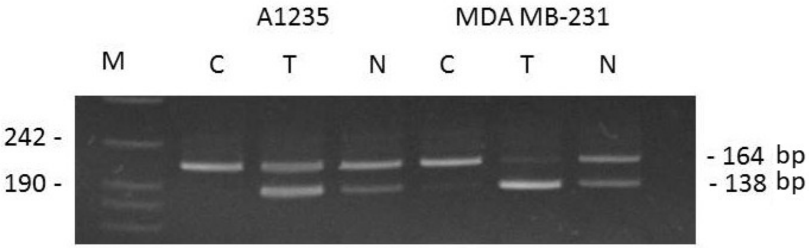

Stained polyacrilamide gel with
PCR products of cDNA amplification
with XBP1 primers
